# Association of a self-efficacy theory-driven doula program combined with stage-focused nursing with delivery outcomes: a retrospective study

**DOI:** 10.3389/fmed.2026.1683230

**Published:** 2026-06-11

**Authors:** Hongyan Xiao, Lijuan Li

**Affiliations:** Department of Obstetrics and Gynecology, Fuyang Women and Children’s Hospital, Fuyang, China

**Keywords:** delivery outcomes, doula, labor pain, self-efficacy, stage-focused nursing

## Abstract

**Objective:**

This retrospective observational study evaluated the association between a doula delivery program based on self-efficacy theory combined with stage-focused nursing and delivery outcomes (postpartum hemorrhage, perineal tears, and neonatal Apgar scores), labor duration, and labor pain. Secondary objectives included assessing associations with fear of childbirth, psychological state, resilience, childbirth self-efficacy, self-control, and childbirth experience.

**Methods:**

This retrospective study analyzed clinical data from 98 parturients who underwent vaginal delivery at the hospital between January 2021 and December 2023. Parturients were divided into a comparison group (*n* = 49, received stage-focused nursing only) and a study group (*n* = 49, received a self-efficacy theory-based doula program combined with stage-focused nursing) based on the nursing protocol documented at admission, with non-randomized group allocation. Data on delivery outcomes, labor duration, pain intensity (visual analog scale, VAS), fear of childbirth (CAQ), psychological state (PANAS), psychological resilience (CDRISC-10), childbirth self-efficacy (CBSEI-C32), self-control (LAS), and childbirth experience (CEQ) were extracted from electronic medical records. Statistical analysis employed independent *t*-tests, chi-squared tests, and repeated-measures analysis of variance (ANOVA) as appropriate.

**Results:**

The study group had a significantly shorter total labor duration (562.76 ± 34.85 vs. 608.59 ± 41.74 min, *p* < 0.001), lower labor pain score (VAS), higher neonatal Apgar score (9.26 ± 0.13 vs. 8.74 ± 0.18, *p* < 0.001), and improved psychological outcomes (CAQ, PANAS, and CDRISC-10).

**Conclusion:**

In this retrospective study, the self-efficacy theory-based doula program combined with stage-focused nursing was associated with improved delivery outcomes, reduced pain, and enhanced psychological wellbeing. However, due to the observational design and potential selection bias, causal inferences cannot be drawn. These observed associations need to be confirmed through prospective randomized controlled trials.

## 1 Introduction

Psychological factors—including fear of childbirth, anxiety, and low perceived control—constitute a major barrier to successful vaginal delivery, which is otherwise the optimal mode of birth for eligible pregnancies. Observational studies suggest that vaginal delivery may be associated with favorable neonatal outcomes, including improved respiratory adaptation and colonization with potentially protective microbiota acquired through maternal birth canal exposure, although the causal pathways remain incompletely understood (1). A cost-effectiveness analysis conducted within the Brazilian Unified National Health System further supports spontaneous vaginal delivery as the economically favorable option for normal-risk pregnancies (2). At the policy level, the World Health Organization (WHO) recommends that the cesarean section rate be controlled at 10–15% to avoid unnecessary medical risks and negative effects on maternal and child health (3). The International Federation of Gynecology and Obstetrics (FIGO) similarly calls for professional organizations to support vaginal delivery, with cesarean section reserved for medically necessary cases; an overview of systematic reviews has identified antenatal and intrapartum interventions that may reduce cesarean section rates and promote vaginal birth (4). Therefore, optimizing support for intended vaginal delivery is a clinical priority.

However, the psychological demands of labor are not adequately met by the current physiological nursing models. A concept analysis of women’s fear of childbirth during pregnancy identifies this fear as a multidimensional phenomenon that can negatively influence birth outcomes (5). Factors such as pain, physical exhaustion, and uncertainty during childbirth often provoke fear, anxiety, and helplessness, with studies among primiparous women confirming that fear and negative childbirth experiences are prevalent and interrelated (6). In 2018, the WHO recommended that women receive a holistic, human rights-based intrapartum care program to relieve negative emotions and promote a positive childbirth experience (7). Stage-focused nursing—a locally developed protocol representing the standard care at our institution—organizes nursing interventions around the three physiological stages of labor. While this approach ensures physiological safety, it is dominated by medical staff and places parturients in a passive role, without systematically addressing psychological determinants of labor progression such as confidence and perceived control (5, 6).

The self-efficacy theory, proposed by Albert Bandura, provides a framework for addressing these psychological determinants. Self-efficacy refers to an individual’s belief in her capacity to execute behaviors necessary to achieve specific outcomes (8). The theory identifies four sources of self-efficacy—mastery experience, vicarious experience, verbal persuasion, and regulation of physiological and emotional states—that can be targeted to enhance coping confidence. In the context of childbirth, self-efficacy denotes a woman’s confidence in her ability to cope with labor and manage pain; a recent cluster analysis has demonstrated that fear of childbirth, anxiety, depression, and childbirth self-efficacy are interrelated constructs that jointly influence maternal outcomes (9). Therefore, interventions designed to systematically enhance self-efficacy through its four theoretical sources may address the psychological barriers inadequately managed through conventional nursing models.

Previous studies have examined self-efficacy-based interventions and stage-focused nursing separately. A randomized controlled trial of childbirth education based on self-efficacy principles reported improved perceptions of childbirth and obstetric outcomes among nulliparous women (10). Additionally, a systematic review and meta-analysis of mindfulness-based interventions found that such approaches were associated with improvements in self-efficacy and reductions in fear of childbirth, although effect sizes varied across study designs (11). However, these studies evaluated psychological interventions or physiological nursing models in isolation. No prior study has integrated a theory-driven doula program explicitly structured around Bandura’s four sources of self-efficacy with stage-focused nursing care.

Conventional doula services offer continuous intrapartum support; however, their content varies widely. A Cochrane systematic review on continuous support for women during childbirth concluded that, while doula support is associated with more favorable outcomes, the components of doula care are inconsistently defined and operationalized across studies (12). Furthermore, research on postpartum doulas indicates that doula practice is often guided by experiential knowledge rather than explicit psychological theory, with motivations and perceptions varying considerably among practitioners (13). Few doula programs specify self-efficacy theory as their guiding framework, limiting their potential to systematically build maternal confidence. Additionally, studies of self-efficacy-oriented doula programs remain scarce in the literature; a recent systematic review of mindfulness-based and self-efficacy-focused interventions for pregnant women noted that the integration of doula support with formal self-efficacy theory has not been adequately investigated (14).

The present study evaluated a combined intervention designed to bridge this gap. The doula program was systematically mapped onto Bandura’s four self-efficacy sources, while stage-focused nursing provided structured physiological support. The rationale for this integration was two-fold: (1) Self-efficacy theory offers a robust framework to reduce maternal helplessness and fear by building coping confidence (8, 9), and (2) stage-focused nursing addresses physiological needs that psychological intervention alone cannot replace. By targeting both domains simultaneously, this combined approach was designed to address the psychological limitations of stage-focused nursing and the theoretical limitations of conventional doula care in a single integrated protocol. No prior study has comprehensively evaluated such a theory-driven, integrated model.

Therefore, this study aimed to evaluate the association between this combined intervention and maternal delivery outcomes and labor analgesia. The primary outcomes comprised delivery-related outcomes (postpartum hemorrhage, perineal tears, and neonatal Apgar scores), the duration of each stage of labor, and labor pain intensity. Secondary outcomes included fear of childbirth, psychological state, resilience, childbirth self-efficacy, self-control, and the childbirth experience.

## 2 Materials and methods

### 2.1 Study participants

This retrospective cohort study analyzed the clinical data of 98 parturients who underwent vaginal delivery at the hospital from January 2021 to December 2023. Data were retrospectively extracted from electronic medical records. A retrospective design was chosen to leverage existing clinical data and real-world practice patterns. (1) Inclusion criteria: Maternal clinical data were included if they met the following criteria: singleton pregnancy, full-term delivery (37–42 weeks), eligibility for vaginal delivery, age 20–35 years, normal coagulation function, and no severe comorbidities. (2) Exclusion criteria: The exclusion criteria were as follows: maternal pelvis abnormalities, birth canal abnormalities, or fetal malformations; cesarean section indications or transfer cesarean section; previous experience of doula-accompanied delivery; postpartum hemorrhage, amniotic fluid embolism, or other serious childbirth complications; newborns needed to be treated in neonatal wards for various reasons after birth; assisted reproductive technology; premature or expired pregnancy; painless childbirth; blood system diseases; or autoimmune system diseases.

No *a priori* power analysis was conducted to determine the required sample size, which is a methodological limitation of this retrospective study. The sample size (*N* = 98) was determined by the available data from the 3-year study period. A *post-hoc* power analysis indicated that this sample size provides 80% power to detect a moderate effect size (Cohen’s *d* = 0.6) for the primary outcome (total labor duration) at a two-sided *α* = 0.05. Although *a priori* sample size was not calculated, the sample size was considered sufficient to detect significant differences on the basis of effect sizes reported in similar studies (15). Based on the nursing protocol documented at admission, participants had self-selected into two groups: a comparison group (received stage-focused nursing only) and a study group (received a self-efficacy theory-based doula program combined with stage-focused nursing). Group allocation was non-randomized and reflected the clinical protocol the parturient chose to receive upon admission, subject to real-time availability of doula services. The study was approved by the Medical Ethics Committee of Fuyang Women and Children’ s Hospital (No. FYBJ2020073). Written informed consent was obtained from all participants at the prenatal visit (1–2 weeks before delivery), at which time the nature of the clinical protocols, including the doula program and the routine collection of psychological assessments at standardized time points, was explained.

To mitigate selection bias and confounding in this non-randomized design, the following strategies were used: (1) Consecutive case inclusion: All eligible parturients admitted during the study period who met the inclusion criteria and had complete data were included, without the researchers selecting cases. (2) Baseline equivalence assessment: Demographic and obstetric characteristics were compared between groups (Table 1) to evaluate covariate balance. (3) Explicit documentation of factors influencing self-selection, including doula staffing availability, cervical dilation at presentation, and patient request, as described above.

**Table 1:** Comparison of general data between the two groups of parturients.

Index	Study group (*n* = 49)	Comparison group (*n* = 49)	Statistic	*p*
Type of maternity [*n* (%)]				
Primiparous	44(89.80)	46 (93.88)	*χ*^2^ = 0.136	0.712
Multiparous	5 (10.20)	3 (6.12)		
Age ( $\bar{x}$ ±*s*, year)	27.12 ± 2.15	27.03 ± 2.11	*t* = 0.209	0.835
Delivery pregnancy week ( $\bar{x}$ ±*s*, week)	39.02 ± 0.71	39.13 ± 0.69	*t* = 0.778	0.439
Height ( $\bar{x}$ ±*s*, cm)	162.51 ± 4.54	162.07 ± 4.46	*t =* 0.484	0.630
Residential arrangement [*n* (%)]				
Couple living alone	14 (28.57)	17 (34.69)	*χ*^2^ = 0.425	0.515
Living with parents	35 (71.43)	32 (65.31)		
Prenatal fetal ultrasound examination of body mass ( $\bar{x}$ ±*s*, g)	3371.34 ± 326.28	3255.02 ± 337.32	*t* = 1.735	0.086
Weight before delivery ( $\bar{x}$ ±*s*, kg)	71.30 ± 2.02	71.48 ± 2.13	*t =* 0.429	0.669
Standard of culture [*n* (%)]				
Junior high school and below	13 (26.53)	15 (30.61)	*χ*^2^ = 0.200	0.655
High school and above	36 (73.47)	34 (69.39)		

### 2.2 Clinical protocols documented in the two groups

The clinical protocols that participants received were documented in the medical records at the time of care. For the purpose of this retrospective analysis, the protocol documented for each participant determined her group classification.

#### 2.2.1 Protocol documented in the comparison group

The stage-focused nursing documented for the comparison group represents the standard labor management protocol routinely used at our institution. It is a locally developed, structured care protocol that divides labor into three stages (first, second, and third), with nursing interventions tailored to the physiological needs and clinical priorities of each stage. This protocol is not based on a specific psychological theory; rather, it reflects conventional intrapartum nursing practice organized around stage-based clinical milestones. The specific components are detailed below. (1) First stage of labor (comfort care): Registered nurses on staff provided a quiet environment with soft mattresses for rest. Position changes (lateral/upright) were conducted every 2 h by the nursing staff to prevent discomfort. Family members were instructed to provide oral hydration and energy supplements every 1–2 h under nurse supervision. (2) Second stage of labor (force guidance): Nurses offered 1:1 force guidance during contractions, with real-time feedback on facial expressions and body posture. Midwifery tools were used as needed and were documented by attending physicians. (3) Third stage of labor (postpartum care): Newborn skin-to-skin contact was initiated within 1 min of delivery by nurses. Maternal vital signs were monitored every 15 min for the first hour by nursing staff.

#### 2.2.2 Protocol documented in the study group

The self-efficacy-based doula program documented for the study group was integrated into routine clinical care. All procedures were documented in the electronic medical records at the time of care. The program was implemented by trained doulas meeting the following criteria: a minimum of 3 years of obstetric nursing experience, completion of a 40-h certified doula training program, and demonstrated competency in labor support techniques. Doulas followed a standardized protocol integrating Bandura’s four sources of self-efficacy: mastery experience, vicarious experience, verbal persuasion, and regulation of physiological and emotional states. The protocol components were explicitly mapped onto these theoretical sources as follows: (1) In the prenatal stage (1–2 weeks before delivery), group education sessions (2 sessions, 90 min each) were conducted using videos, models, and case sharing (vicarious experience). Individualized delivery plan development was documented in medical records (verbal persuasion and mastery experience). (2) In the intrapartum stage (from admission to 2 h postpartum), pain management included the Doula Analgesia Instrument (GT-4A model, current 0.1–0.3 mA) applied when cervix dilated to 3 cm, used until full dilation (regulation of physiological states). According to the manufacturer’s technical documentation, this device uses transcutaneous electrical nerve stimulation (TENS) technology to inhibit pain signal transmission from the spinal cord to the central nervous system. The GT-4A device is a commercially available TENS unit; however, peer-reviewed validation studies specifically evaluating its efficacy for labor analgesia were not identified. In this study, the device was applied as part of the standard doula protocol at our institution. Its contribution to the observed between-group differences in pain scores cannot be isolated from the other concurrent interventions, and it may constitute a confounding therapeutic modality.

Pain management through breathing and massage: This entailed guiding the use of prenatal breathing techniques during contractions while providing massage to the lower back, abdomen, and sacrococcygeal region (regulation of physiological states and mastery experience). Doula pain relief device: This device was used when the cervix dilated to 3 cm (GT-4A model, current 0.1–0.3 mA) until full dilation. Perineal heat therapy: A 50% magnesium sulfate solution heat pack was applied during the contractions for 10 min per session (regulation of physiological states). Music intervention: The mother’s preferred soothing music was played to distract her (regulation of emotional states). Autonomy support: The mother was permitted to choose her preferred birthing position (e.g., side-lying, semireclining, and kneeling) and told how the position could facilitate labor progression (verbal persuasion and mastery experience). The mother was encouraged to express her needs at any time (e.g., adjusting the lighting, and adding or removing clothing) (verbal persuasion). (3) Postpartum stage: The mother was given immediate feedback and emotional reinforcement (verbal persuasion). She had immediate skin-to-skin contact with the newborn, whereas staff simultaneously acknowledged her mother’s efforts (e.g., ‘You welcomed the baby with your own strength’) (verbal persuasion and mastery experience). The key points of the labor process (e.g., strategies for managing contractions) needed to reinforce a sense of self-efficacy were reviewed (mastery experience). Postpartum recovery and psychological guidance: Staff explained the postpartum diet, rest, and breastfeeding techniques, monitored the mother’s psychological state, and guided her to adopt a positive attitude toward postpartum changes (verbal persuasion and regulation of emotional states).

The availability of the doula-led protocol for an individual patient upon admission was influenced by several factors: (1) doula staffing availability at the time of admission, as doula services were not available 24 h per day; (2) the patient’s explicit verbal request for doula support upon admission, which was then documented in the medical record; (3) the patient’s cervical dilation at presentation, as the doula protocol was typically initiated when dilation was ≤4 cm, allowing sufficient time for the full intrapartum protocol to be implemented. There were no restrictions based on parity, singleton/twin status (only singletons were included per inclusion criteria), or time of day, other than staffing limitations. All participants in both groups were singleton pregnancies by inclusion criteria.

### 2.3 Data collection

Demographic and outcome data were retrospectively extracted from electronic medical records by two independent researchers. All psychological scales and the VAS were administered as part of routine clinical care by trained nurses at standardized time points and documented in the medical records prior to the study’s inception. Specifically, the CAQ, PANAS, CDRISC-10, and CBSEI-C32 were administered at the prenatal visit (1–2 weeks before delivery) and at 48 h postpartum. The LAS and CEQ were administered at 48 h postpartum only. VAS pain scores were recorded by nursing staff at the end of each of the three stages of labor. The completion rate for all administered instruments was 100% for the 98 participants included in this analysis as complete data at both time points were an implicit requirement for inclusion; participants with missing questionnaire data were not included in the study dataset.

Labor stage durations were abstracted from nursing flow sheets, pain scores from VAS records, and psychological scales from documented assessments at prenatal visits and 48 h postpartum. Discrepancies were resolved by a third reviewer. (1) Delivery outcome. The vaginal midwifery rate, 2-h postpartum bleeding volume, total labor time, perineal tear rate, and Apgar score at 5 min after birth were compared between the two groups (16). (2) Different labor times and degrees of pain. The first, second, and third stages of labor were compared between the two groups. At the end of each of these stages, the VAS (17) was used to measure labor pain, which directly reflects the analgesic effect of the Doula Analgesia Instrument and relaxation training. The VAS (18) was used to evaluate the degree of labor pain in the two groups. The scale is composed of a 0–10 cm straight line (0–10 points, corresponding to completely painless to most painful). Guiding the parturient to choose the corresponding position of the straight line can enable her to indicate the pain level she is experiencing at that time. The higher the score is, the greater the degree of pain is. The Cronbach’s *α* coefficient of the VAS in this study was 0.904. (3) Fear of childbirth and psychological state. 1. Fear of childbirth: 48 h before and after delivery, the CAQ scale (19) assessed childbirth fear, which corresponds to the intervention’s goal of reducing anxiety through self-efficacy enhancement. The CAQ scale (20) was used to evaluate the fear of childbirth. The scale includes 16 items in four dimensions, namely, fear of pain injury (7/8/12/13, 4 items in total), fear regarding child health (4/6/9/10/11, 5 items in total), fear regarding willingness to intervene and the environment (3/14/15, 3 items in total), and fear of losing control during childbirth (1/2/5/16, 4 items in total). Each item is scored on a 4-point Likert scale (none to high, 1–4 points, respectively). The scale score ranged from 16 to 64 points, with 16 points ≤ score ≤ 27 points, 28 points ≤ score ≤ 39 points, 40 points ≤ score ≤ 51 points, and 52 points ≤ score ≤ 64 points, representing no, mild, moderate, and severe fear of childbirth, respectively. The Cronbach’s *α* coefficient of the CAQ scale in this study was 0.910. 2. Psychological status: 48 h before and after delivery, Watson et al. (21) and Huang et al. (22) used the PANAS. The scale consists of two subscales: positive emotion (PA) and negative emotion (NA). Each subscale consists of 10 items. Each item is scored on a 5-point Likert scale (almost no to very many, 1–5 points, respectively). The PA subscale score ranged from 10 to 50 points (the higher the score is, the more positive the psychological state is), and the NA subscale score ranged from 10 to 50 points (the higher the score is, the more negative the psychological state is). The Cronbach’s *α* coefficients of the PANAS scale, PA subscale, and NA subscale in this study were 0.926, 0.937, and 0.919, respectively. (4) Resilience and childbirth self-efficacy. 1: The psychological resilience scale (CDRISC-10) compiled by Campbell-Sills et al. (23) and translated and revised by Wang et al. (24) was used to evaluate psychological resilience before and 48 h after delivery. The scale contains 10 items, and each item is scored on a 5-point Likert scale (never to almost always, 0–4 points). The scale score ranged from 0 to 40 points. The higher the score is, the greater the degree of psychological resilience is. The Cronbach’s *α* coefficient of the CDRISC-10 scale in this study was 0.885. 2. Delivery self-efficacy: Before delivery and 48 h after delivery, the simplified version of the delivery self-efficacy scale (CBSEI-C32) developed by Lowe (25) and by Ip et al. (26) was used to evaluate delivery self-efficacy. The scale includes two subscales: outcome efficacy (OE-16) and expected efficacy (EE-16), with a total of 32 items. Each subscale has 16 items, and each item is scored on a 10-point Likert scale. On the OE-16 subscale (score 16–160 points), each item is completely unhelpful–very helpful (1–10 points, respectively). On the EE-16 subscale (score 16–160 points, each item is rate on a 10-point scale, completely negative to completely negative is counted as 1–10 points), the higher the score is, the greater the self-efficacy is. In this study, the Cronbach’s *α* coefficients of the CBSEI-C32 scale, OE-16 subscale, and EE-16 subscale were 0.923, 0.921, and 0.926, respectively. (5) Delivery self-control, delivery experience, and postpartum lactation function. 1 Self-control of childbirth: 48 h after delivery, the childbirth control scale (LAS) compiled by Hodnett et al. (27) and applied by Gau et al. (28) was used to evaluate the self-control of childbirth. There are 29 items in the scale, and each item is scored on a 7-point scale (strongly agree to strongly disagree), 14 items are scored positively, 15 items are scored negatively, and the scale score ranged from 29 to 203. The higher the score is, the greater the self-control of childbirth is. The Cronbach’s *α* coefficient of the LAS scale in this study was 0.872. 2. Delivery experience: 48 h after delivery, the childbirth experience scale (CEQ) developed by Dencker et al. (29) and translated and adapted by Zhu et al. (30) was used for evaluation. The scale includes 19 items in four dimensions, namely, sense of participation (10/11/12, a total of 3 items), self-perception (3/5/8/9, a total of 4 items), self-ability (1/2/4/6/7/19, a total of 6 items), and professional support (13/14/15/16/17/18, a total of 6 items). Each item is scored on a 4-point Likert scale (strongly disagree–strongly agree, 1–4 points, respectively), in which the items of the self-perception dimension are scored in the opposite direction and the items of the other dimensions are scored in the positive direction. The total score of the scale = the score of all the items/the total number of items, and the score of the scale ranged from 1 to 4 points. The higher the score is, the better the delivery experience is. In this study, the Cronbach’s *α* coefficient of the CEQ scale was 0.802.

### 2.4 Statistical analysis

SPSS 23.0 software (IBM, Armonk, New York, USA) was used. The normality of continuous variables was tested using the Shapiro–Wilk test. For normally distributed data, independent samples *t*-tests or repeated-measures ANOVA were applied; otherwise, non-parametric tests (e.g., the Mann–Whitney *U* test) were used. For repeated-measures data (e.g., labor stage durations), repeated-measures ANOVA was performed. For normally distributed pain scores (VAS scores), paired *t*-tests were used; non-normally distributed data were analyzed via the Wilcoxon signed-rank test, and the count data were expressed as *n* (%). A *p-*value of < 0.05 was considered statistically significant. For multiple comparisons, the Bonferroni correction was not applied because of the prespecified primary outcomes (labor duration and pain scores); however, effect sizes (Cohen’s d) were calculated to assess clinical significance.

## 3 Results

### 3.1 Descriptive characteristics

The baseline characteristics of pregnant women in the comparison group (*n* = 49) and the study group (*n* = 49) are shown in Table 1. For all measured baseline variables, no statistically significant differences were observed between groups (all *p* > 0.05). However, in this non-randomized observational design, the absence of significant differences on measured covariates does not establish group equivalence or balance; residual confounding by unmeasured factors (e.g., baseline motivation and childbirth anxiety) may persist. Although most baseline characteristics were comparable, the between-group difference in prenatal fetal ultrasound weight (*p* = 0.086) approached significance, with participants in the study group having slightly larger fetuses (3,371 ± 326 g vs. 3,255 ± 337 g). This imbalance, if anything, would bias the results toward the null hypothesis as larger fetal size is typically associated with more difficult labor.

### 3.2 Delivery outcomes

Compared with the comparison group, the study group had fewer 2-h postpartum hemorrhages, fewer cases of vaginal midwifery and perineal tears, shorter total labor times, and higher 5-min Apgar scores (Cohen’s *d* = 3.35) for newborns (*p* < 0.05) (Table 2).

**Table 2:** Comparison of delivery outcomes between the two groups.

Group	Postpartum blood loss after 2 h ( $\bar{x}$ ±*s*, mL)	Neonatal 5-min Apgar score ( $\bar{x}$ ±*s*, score)	Total duration of labor ( $\bar{x}$ ±*s*, min)	Vaginal delivery [*n* (%)]	Laceration of perineum [*n* (%)]
Study group (*n* = 49)	109.41 ± 8.52	9.26 ± 0.13	562.76 ± 34.85	2 (4.08)	5 (10.20)
Comparison group (*n* = 49)	126.53 ± 9.15	8.74 ± 0.18	608.59 ± 41.74	9 (18.37)	14 (28.57)
*t*/*χ*^2^	−9.585	16.394	−5.900	5.018	5.289
*p*	<0.001	<0.001	<0.001	0.025	0.022

### 3.3 Different labor times and degrees of pain

Repeated-measures ANOVA was used to analyze the labor stage durations and VAS scores, revealing significant group effects for all the stages (first stage: *F* = 16.85, *p* < 0.001; second stage: *F* = 22.47, *p* < 0.001; third stage: *F* = 31.52, *p* < 0.001). *Post-hoc* tests revealed shorter durations and lower VAS scores in the study group (all *p* < 0.05). The Shapiro–Wilk test confirmed the normal distribution of the VAS scores (*W* = 0.91, *p* = 0.13) (Table 3).

**Table 3:** Comparison of labor stage duration and VAS score using repeated-measures ANOVA ( $\bar{x}$ ±*s*).

Group	Different duration of labor (min)			VAS score of different stages of labor		
	First stage of labor	Second stage of labor	Placental stage	First stage of labor	Second stage of labor	Placental stage
Study group (*n* = 49)	498.79 ± 31.85	51.34 ± 5.12	12.63 ± 1.74	3.42 ± 0.35	6.28 ± 0.54	3.16 ± 0.27
Comparison group (*n* = 49)	526.83 ± 35.62	62.45 ± 7.83	19.31 ± 2.08	4.17 ± 0.41	7.39 ± 0.62	3.83 ± 0.34
*F/t*	16.85	8.313	17.243	22.47	9.450	10.802
*p*	<0.001	<0.001	<0.001	<0.001	<0.001	<0.001

### 3.4 Fear of childbirth and mental state

Compared with those of the prenatal group, the CAQ score and the NA subscale score on the PANAS scale of the two groups were lower at 48 h after delivery, whereas the PA subscale score on the PANAS scale was higher (*p* < 0.05). Compared with those in the comparison group, the CAQ score and the NA subscale score on the PANAS scale in the study group were lower, and the PA subscale score on the PANAS scale was higher (*p* < 0.05) (Table 4).

**Table 4:** Comparison of CAQ scores and PANAS subscale scores before and 48 h after delivery between the two groups ( $\bar{x}$ ±*s*, score).

Group	CAQ score		PANAS scale PA subscale score		PANAS scale NA subscale score	
	Ante partum	48 h after delivery	Ante partum	48 h after delivery	Ante partum	48 h after delivery
Study group (*n* = 49)	40.45 ± 3.71	25.39 ± 1.62^*^	25.49 ± 2.64	41.37 ± 3.36^*^	38.34 ± 3.45	20.21 ± 1.57^*^
Comparison group (*n* = 49)	41.03 ± 3.86	30.43 ± 2.29^*^	26.03 ± 2.68	35.38 ± 3.09^*^	37.89 ± 3.51	24.62 ± 2.34^*^
*t*	0.758	−12.577	−1.005	9.185	0.640	10.955
*p*	0.450	<0.001	0.318	<0.001	0.524	<0.001

Compared with the group before delivery, *p < 0.05.

### 3.5 Resilience and childbirth self-efficacy

Compared with those of the prenatal group, the scores of the CDRISC-10, OE-16 subscale, and EE-16 subscale of the CBSEI-C32 scale in the two groups were greater at 48 h after delivery (*p* < 0.05), and the above scores in the study group were greater than those in the comparison group (*p* < 0.05) (Table 5).

**Table 5:** Comparison of CDRISC-10 scores and CBSEI-C32 subscale scores between the two groups before and 48 h after delivery ( $\bar{x}$ ±*s*, score).

Group	CDRISC-10 score		CBSEI-C32 scale OE-16 subscale score		CBSEI-C32 scale EE-16 subscale score	
	Ante partum	48 h after delivery	Ante partum	48 h after delivery	Ante partum	48 h after delivery
Study group (*n* = 49)	21.23 ± 2.41	33.72 ± 3.69^*^	85.31 ± 4.78	115.72 ± 5.84^*^	89.26 ± 4.87	118.32 ± 6.06^*^
Comparison group (*n* = 49)	21.39 ± 2.48	28.26 ± 3.17^*^	86.02 ± 4.83	104.63 ± 5.09^*^	89.87 ± 4.94	107.41 ± 5.35^*^
*t*	0.324	7.857	0.731	10.021	0.616	9.447
*p*	0.747	<0.001	0.466	<0.001	0.540	<0.001

Compared with the group before delivery, *p < 0.05.

### 3.6 Sense of childbirth self-control and childbirth experience

Compared with the comparison group, the study group had higher LAS scores (173.78 ± 9.25 vs. 158.32 ± 6.64, *p* < 0.001), indicating greater self-control during childbirth according to the scale’s scoring direction and higher CEQ scores (3.31 ± 0.26 vs. 2.86 ± 0.23, *p* < 0.001) at 48 h postpartum (Table 6). The LAS scores indicated greater self-control in the study group, consistent with the scale’s scoring direction (higher scores = greater control).

**Table 6:** Comparison of the LAS score, CEQ score, and postpartum lactation function between the two groups at 48 h after delivery.

Group	LAS score ( $\bar{x}$ ±s, score)	CEQ score ( $\bar{x}$ ±s, score)
Study group (*n* = 49)	173.78 ± 9.25	3.31 ± 0.26
Comparison group (*n* = 49)	158.32 ± 6.64	2.86 ± 0.23
*t*/*χ*^2^	9.504	9.074
*p*	<0.001	<0.001

## 4 Discussion

The delivery process is a dual test of maternal physical and psychological quality. Severe labor pain, fear of childbirth, and the psychological stress response caused by childbirth have become the main stressors affecting maternal delivery status. These stress responses can increase the degree of maternal sympathetic nerve excitation, promote the release of stress hormones such as catecholamines and adrenaline, inhibit uterine vasoconstriction and uterine contraction, disrupt uterine contraction coordination, and prolong labor (31). The following discussion of potential mechanisms is speculative and intended to generate hypotheses for future research as the current study design does not permit causal or mechanistic conclusions. Bandura’s self-efficacy theory identifies four core sources of self-efficacy: direct experience, vicarious experience, verbal persuasion, and physiological/emotional states. The doula program systematically integrated these sources: Direct experience, prenatal respiration, and relaxation training allowed parturients to practice coping strategies, building confidence through repeated successful simulations. Vicarious experience: Postpartum case sharing sessions with women who delivered successfully naturally provided observational learning, reducing fear through learning about others’ experiences. Verbal persuasion: Doulas provided continuous affirmation during labor (e.g., “You are managing well”), reinforcing self-efficacy beliefs. Physiological regulation: The Doula Analgesia Instrument and perineal hot compresses mitigated pain-induced physiological stress, preventing efficacy erosion from negative emotional states. Carrying out nursing measures around self-efficacy theory may be associated with improving the outcomes of childbirth, which merits further exploration (32). It is important to note that, although self-efficacy measures improved in the study group, no mediation analysis was performed to establish a statistical association between changes in self-efficacy and clinical outcomes. Therefore, the theoretical interpretation that observed clinical benefits were mediated by self-efficacy enhancement remains speculative and requires confirmation in future studies designed for causal mediation analysis.

The results of this study are consistent with those of Zhang et al. (33) and Çankaya et al. (34). However, it is important to note that those studies employed prospective randomized designs, whereas our study is retrospective and observational. The consistency of findings across different study designs strengthens the evidence base; however, the methodological differences require cautious interpretation of causal relationships. The combined nursing program was associated with improved maternal self-efficacy, sense of delivery control, and childbirth experience and was associated with lower CAQ scores. The reasons for this may be as follows: (1) Self-efficacy can be improved through direct experience, alternative experience, verbal persuasion, physiological emotion regulation, and other channels. On the basis of the theory of self-efficacy, the design of alternative experiences, verbal persuasion, and physiological emotion regulation in the construction of a doula delivery program is helpful for systematically improving maternal self-efficacy (35). Among these strategies, it is possible that prenatal use of videos, models, and other intuitive approaches to explain natural childbirth, the characteristics of different stages of labor, and corresponding coping measures to mothers and their families can help mothers build a preliminary cognitive framework for natural childbirth, have a clear blueprint for childbirth in their minds, eliminate the fear of the unknown, and increase their confidence in dealing with the unknown. Meetings where mothers share stories of recent successful spontaneous delivery can inform pregnant women of the key content of the delivery process, such as personal experience, psychological transformation, and coping strategies, so that they can have alternative experiences, increase their confidence through hearing about others’ successful experience, and help them believe that they have the same ability (36). Through in-depth communication with the puerpera, we can create a personalized delivery plan and post it in a prominent position for the puerpera to view and review at any time. It is helpful for medical staff to understand the needs of the puerpera and ensure the effective implementation of the delivery plan. This can also help the puerpera feel in control of the delivery process and increase their self-efficacy. Prenatal respiratory and relaxation training can help parturients skilfully use breathing to regulate body tension so that they can learn to relax quickly during childbirth, reduce negative emotions, and gradually strengthen their skills and confidence in dealing with uterine contraction pain (37). (2) Providing continuous companionship and psychological support for pregnant women during the delivery stage, affirming the efforts of pregnant women, can help pregnant women stabilize their emotions, restore confidence in smooth delivery, and strengthen self-efficacy beliefs. Under the premise of complying with medical norms, medical staff can assist patients in choosing their own delivery positions, explain the role of each position, affirm the efforts of the patient, encourage them to express their needs at any time according to their physical feelings, and enable the patient to realize the positive impact of their actions on the delivery results. Pregnant women can be enabled to feel that they are the leaders of childbirth rather than passively receiving medical treatment (38). (3) In the postpartum stage, the newborn should have contact with the skin of the mother immediately after the baby is delivered. The mother should be warmly congratulated on her strength to usher in this healthy life, which can sublimate her sense of achievement and self-efficacy in childbirth, help her accumulate confidence in the face of other challenges in the future, and improve her psychological resilience. Gentle assistance with maternal placenta delivery, perineal suturing, and other follow-up procedures can alleviate maternal anxiety and ensure the smooth progress of postpartum treatment. Importantly, although self-efficacy measures improved in the study group, no mediation analysis was performed to establish a statistical association between changes in self-efficacy and clinical outcomes. Therefore, the theoretical interpretation that observed clinical benefits were mediated by self-efficacy enhancement remains speculative and requires confirmation in future studies designed for causal mediation analysis.

The results of this study indicated that the delivery outcomes of the study group were better than those of the comparison group after nursing. The first, second, and third stages of labor were shorter, and the VAS scores of the different stages of labor were lower. The difference in the neonatal Apgar scores (0.52 points, Cohen’s *d* = 3.35) yielded an unadjusted effect size estimate that is larger than typically observed in clinical research. This magnitude should be interpreted with caution, as this unadjusted comparison does not account for potential confounding or selection bias, and the observed difference may substantially overestimate the true association. The clinical significance of a 0.52-point difference, while arising from the data, requires validation in prospective studies before being considered clinically meaningful. Similarly, the 45.83-min reduction in total labor time (Cohen’s *d* = 1.21) aligns with clinical standards for meaningful labor acceleration. Similar to the conclusions of Sun et al. (39), the combined nursing program may be beneficial through its association with the duration and degree of pain of each stage of labor, promotes lactation, and improves delivery outcomes. The reasons for this difference may be related to the following points: (1) Playing mothers’ favorite music or soothing music during labor can shift maternal attention to pain, adjust breathing and heart rate, relieve fatigue, restore physical strength, and help them face childbirth in a relatively relaxed state and is associated with the duration of labor (40). The Doula Analgesia Instrument uses continuous activation technology to inhibit the impulse conduction of spinal cord thalamic tract cells, weaken or block the pain information transmitted from the spinal cord to the central nervous system, and effectively reduce pain in the first stage of labor. In the second stage of labor, the perineal hot compress is provided to the patient in the uterine contraction gap, which can promote blood circulation in the perineum. The warm effect of a hot compress can relax the muscles, relieve muscle spasm, reduce the pain caused by fetal compression and perineal dilatation (41, 42), and optimize delivery outcomes. (2) Medical staff should patiently explain postpartum physical recovery precautions to mothers and their families, guide mothers, help mothers increase their confidence in taking care of their babies and their babies’ self-efficacy, and guide mothers to view postpartum changes with a positive attitude. These actions may play a role in psychological adjustment and may help mothers maintain the strong self-efficacy established in the delivery process and better adapt to their new role.

However, several limitations must be acknowledged. First, due to non-randomized self-selection and the absence of multivariable adjustment (constrained by sample size), causal inference is precluded, and residual confounding from unmeasured factors (e.g., motivation and anxiety) cannot be excluded. Second, no *a priori* power calculation was performed; the available sample (*n* = 98) may be underpowered for smaller effects or subgroup analyses. Third, the intervention comprised a bundled set of components (education, massage, music, positioning autonomy, electrical analgesia, heat therapy, and coaching), preventing isolation of self-efficacy enhancement from non-specific support effects. Moreover, the comparison group received structured stage-focused nursing, making the contrast between two active care models, which may have attenuated observed associations. Finally, all comparisons were unadjusted; baseline covariate comparability does not eliminate the risk of bias from unmeasured confounders.

## 5 Conclusion

In summary, in this retrospective observational study, the combined nursing intervention was associated with several improved clinical outcomes, shorter first stage of labor, lower incidence of postpartum hemorrhage, and higher neonatal Apgar scores. However, owing to the retrospective design and potential biases, these findings should be interpreted as hypothesis-generating rather than definitive. These observed associations need to be confirmed through prospective randomized controlled trials.

## Data Availability

The original contributions presented in the study are included in the article/supplementary material, further inquiries can be directed to the corresponding author.
